# A High Precision Quality Inspection System for Steel Bars Based on Machine Vision

**DOI:** 10.3390/s18082732

**Published:** 2018-08-20

**Authors:** Xinman Zhang, Jiayu Zhang, Mei Ma, Zhiqi Chen, Shuangling Yue, Tingting He, Xuebin Xu

**Affiliations:** 1 School of Electronics and Information Engineering, MOE Key Lab for Intelligent Networks and Network Security, Xi’an Jiaotong University, Xi’an 710049, China; zhangxinman@mail.xjtu.edu.cn (X.Z.); mamei19960602@stu.xjtu.edu.cn (M.M.); baimmi.czq@stu.xjtu.edu.cn (Z.C.); jerrytom@stu.xjtu.edu.cn (S.Y.); sixiebb@stu.xjtu.edu.cn (T.H.); 2 Guangdong Xi’an Jiaotong University Academy, No. 3, Daliangdesheng East Road, Foshan 528000, China; ccp9999@126.com

**Keywords:** machine vision, steel bars, quality inspection, dimensional measurement, number counting, high precision, video data acquisition

## Abstract

Steel bars play an important role in modern construction projects and their quality enormously affects the safety of buildings. It is urgent to detect whether steel bars meet the specifications or not. However, the existing manual detection methods are costly, slow and offer poor precision. In order to solve these problems, a high precision quality inspection system for steel bars based on machine vision is developed. We propose two algorithms: the sub-pixel boundary location method (SPBLM) and fast stitch method (FSM). A total of five sensors, including a CMOS, a level sensor, a proximity switch, a voltage sensor, and a current sensor have been used to detect the device conditions and capture image or video. The device could capture abundant and high-definition images and video taken by a uniform and stable smartphone at the construction site. Then data could be processed in real-time on a smartphone. Furthermore, the detection results, including steel bar diameter, spacing, and quantity would be given by a practical APP. The system has a rather high accuracy (as low as 0.04 mm (absolute error) and 0.002% (relative error) of calculating diameter and spacing; zero error in counting numbers of steel bars) when doing inspection tasks, and three parameters can be detected at the same time. None of these features are available in existing systems and the device and method can be widely used to steel bar quality inspection at the construction site.

## 1. Introduction

With the development of modern society, the demand for reinforced concrete in the process of urbanization is greatly increasing, which gives rise to higher technology requirements. In order to ensure the quality of construction projects as well as the safety of builders and occupants, it is urgent to detect whether the steel bars in the building meet the specifications or not.

In urban construction, there are many problems, such as rough construction, thin steel bars and so on, which can seriously affect the building quality and safety [1]. There are three main reasons for this: firstly, the relevant building companies can gain huge economic benefits from shoddy work and sub-standard materials. Secondly, the construction management is lax. Thirdly, the construction workers and the public have poor security awareness. These problems lead to the construction quality failing to meet the acceptance criteria, posing a security risk. The statistics of the Ministry of Housing and Urban-Rural Development of the China (MOHURD) show that from 2004 to 2017 structural collapses occurred a total of 1321 times, accounting for 13.42% of the total number of safety accidents and the number of deaths was 1764, accounting for 19.28% of the total number of deaths from 2004 to 2013. Structural collapses are the leading cause for death among safety accidents and the second most likely cause of safety accidents. Therefore, solving the problem of steel bar quality detection has a profound and important practical significance.

In order to put forward an automated inspection device and method, we searched the domestic and foreign literature about the quality inspection of steel bars [2,3,4,5,6,7,8,9,10,11,12] to know the various detection methods widely used in recent years. Moreover, we organized them into the following Table 1 according to the content of the articles including measuring parameters, sensors employed, inspection method, absolute error, and relative error.

From Table 1, we can summarize that there are four main methods to detect the quality of steel bars, including the measurement of the diameter and spacing, for which most proposed existing methods use static image processing. The first approach is based on machine vision, which has a comparatively high accuracy (as low as 0.072 mm (absolute error) and 0.18% (relative error)), however, it can only calculate the diameter of steel bars through processing static images. The second method is to use ground-penetrating radar (GPR) technology to detect buried matters with different dielectric constant and conductivity. It can calculate the diameter and spacing of steel bars and has a lower accuracy than machine vision-based methods. In addition, GPR equipment is expensive, and it is mainly used to detect the nature and location of underground materials, so it is so wasteful and less precise to measure steel bars diameters in buildings or at the construction site. The third technique is to use steel bar detectors. Currently, most steel bar detection devices use electromagnetic induction to detect steel bars, but such devices are unable to work under a strong alternating electromagnetic field. There is another method using a special neural network to extract the diameter and the spacing of the steel bars. This neural network is trained by data collected from induction sensors. However, the neural network method is complex and can only process static images. It takes a long time cost to train the network although it has a low error. The fourth way is use microwaves to measure the diameter but the error is rather high (5.6 mm (absolute error) and 20% (relative error)). These four methods are all used to detect steel bars embedded in buildings, but none if the existing method focus on the number of steel bars.

Our current goal is to measure the diameter, spacing, and number of steel bars at the same time. Traditional steel bar quality inspection methods mainly utilize Vernier calipers or other fast inspection rulers to detect bars. These methods rely on human eyes for judgement, and they have the disadvantages of large workload, low efficiency, poor accuracy and only sampling small batches at a time, so the results are usually unsatisfactory. In a word, there is essentially no especially efficient method for quality inspection of steel bars in the present construction industry field. The entire construction industry has a huge gap in this area, therefore, an automated, sophisticated inspection device is the best choice. Therefore we focus the method based on machine vision.

Additionally, we can find that the accuracy of machine vision-based detection methods is much higher than that of other methods. These methods have the advantages of real-time and large-capacity detection. Related research is however rare, so we innovatively choose this method to realize steel bar quality inspection. In order to realize the machine vision-based quality inspection of large quantities of steel bars, it is necessary to acquire image and video data of the steel bars in the field. In view of the complex and changeable construction environment, poor working environment and large interference of wireless signals, we have designed a data acquisition device. The device obtains high-precision photos and videos taken by the uniform and stable smartphone in the banding field, then utilizes computer image processing technology to achieve large volume quality detection of several kinds of steel bars in the buildings, and further promotes the construction safety to an unprecedented new level. We conducted multiple tests at the construction site of a power pipe gallery. The acquisition image is clear and the device has a high adjustment accuracy, so the implementation process is effective, which demonstrates that the device and method can be extended to steel bar quality inspection at other construction site in the future.

In this paper, a high precision machine-vision-based inspection system for steel bar quality has been developed. On the construction site, steel bar images or video could be taken by a smartphone, and a practical APP installed on the smartphone would process the video in real-time. Ultimately, the application could show the inspection results which are helpful to users. The rest of the paper is organized as follows: Section 2 describes the principle of the steel bar quality inspection system in detail, including design ideas, the overall architecture, and workflow. Section 3 describes out the methods that are used to detect the steel bars’ diameter, spacing and quantity, including the sub-pixel boundary location method (SPBLM) and fast stitch method (FSM). Section 4 describes the hardware and software components of the steel bar quality inspection system. In Section 5, the applications of this system is discussed and the result analysis is given. Finally, the paper is concluded in the last section.

## 2. Principle of the Steel Bar Quality Inspection System

Due to complexity of the construction environment, different data acquisition and process methods are required in various working environments [13]. It is not easy to realize data acquisition at construction sites, for example, a power pipe gallery. Aiming at solving these problems, we have successfully developed a set of data acquisition equipment and proposed a corresponding method to inspect the quality of steel bars, including the diameter, spacing, and quantity of steel bars.

**Figure 1:**
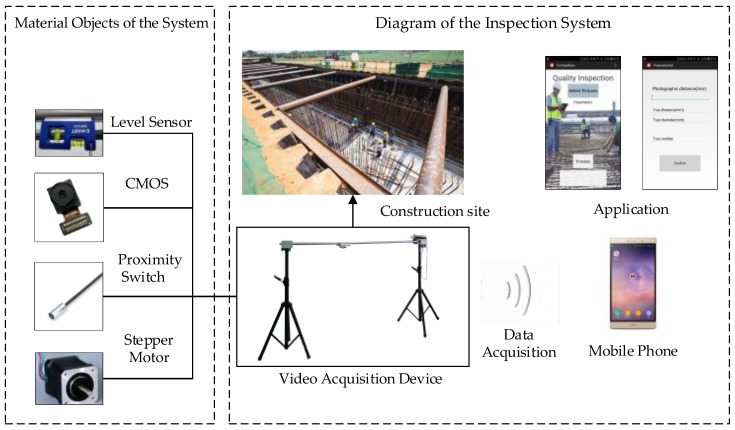
Schematic diagram of video data acquisition system.

A schematic diagram of the data acquisition system is shown as Figure 1. It expresses that a smartphone is installed in the device that under the correct operation conditions captures images or videos from the construction site. The whole device (including the smartphone) uses the following sensors: a level sensor, a CMOS, a proximity switch, a voltage sensor, and a current sensor (the voltage sensor and the current sensor are encapsulated in the power supply and stepper motor). Then data is transmitted to computer in a wireless way or will be processed by a practical APP installed on the smartphone that we specially designed. The acquisition image is clear and the device has high adjustment accuracy, which makes implementation process effective. The smartphone or computer will finish the quality inspection through video processing tasks based on machine vision, including the detection of diameter, spacing, and quantity of steel bars. Finally, the inspection results can be reported to the developers or quality inspection agencies through the application. Once the results are assessed, the developers or quality inspection agencies can immediately order the construction workers to stop watering cement and perform the necessary rework for removing any unqualified steel bars. This system is not complicated, but it can provide sufficient and real-time solutions to the current problems in the construction industry field, greatly ensuring the safety of buildings and personnel.

**Figure 2:**
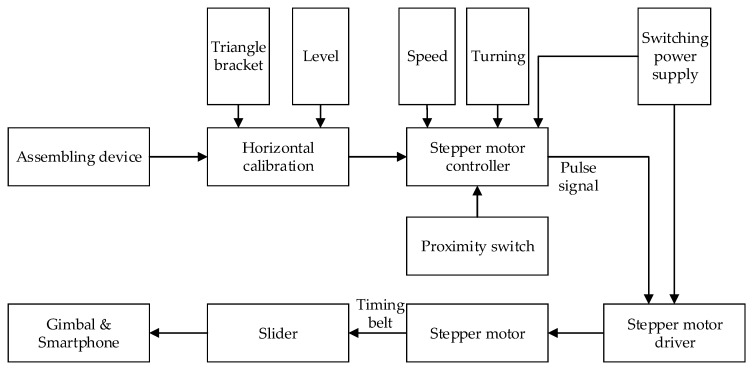
Workflow of data acquisition.

As shown in Figure 2, the video data acquisition process can be described as follows: it is a complete assembled data acquisition device. After arriving at the building site, the components of the equipment are assembled. Then the horizontal calibration operation is carried out. More concretely, the triangular bracket is provided with a hand-wheel capable of controlling the elevation. The horizontal calibration device, level sensor, is mounted on the triangular brackets and the guide rail. The height of the triangular bracket at both ends of the guide rail is adjusted to be equal by rolling the hand-wheel and the guide rail is horizontal. After adjusting the position in place, we set the velocity and turning direction by using the stepping motor controller. Then the stepping motor driver will receive the signal and the motor will drive the slider to move smoothly. At this moment, the mobile phone installed on a gimbal will move to capture images or video and the acquisition process begins. The shooting angle of the gimbal and smartphone are arranged in advance, and the shot is finished when the slider touches the proximity switch at both ends of the guide rail.

## 3. Methods

### 3.1. Image Pretreatment Method 

It’s common that the laying environment of steels is very bad, causing a variety of interference signals in the obtained image. Hence, it is necessary to do de-noising operations in order to improve the quality of images. Afterwards comparing with Wiener filter and mean filter, the median filter has been chosen to decrease the salt and pepper noise on the surface of steels.

Owing to the high precision of steel dimension calculations, the background and target should be segmented in the image. Typically, for purpose of getting a threshold, different methods to solve segmentation problems are proposed. One of the most common and effective methods is the Otsu [14] method, compared with the two-peak method and iterative method, which can get the details more precisely, thereupon making edge detection possible.

The Canny edge detector is an edge detection operator that uses a multi-stage algorithm to detect a wide range of edges in images. The boundary determined by the Canny operator is a single pixel edge, which reflects the distribution of transverse rib edges more precisely.

We conducted four sets of experiments under different illumination and background, and they are shown in Table 2, respectively. We captured images in a laboratory (Group 1) and an outdoor environments, including a normal outdoor environment (Group 2) and a construction site (Groups 3 and 4). For Group 1, the image has a large contrast between foreground (FG) and background (BG) and finally we get clear edges for subsequent processing. In Group 2, the images have a relatively large FG and BG contrast, but the BG is more complex. Although the outline of the reinforcement is a bit defective, we finally get distinct edges and it can be used for subsequent treatment. In Group 3, the image has a totally different illumination and interference of banding knots but we finally get clear edges for post-processing. In Group 4, the FG and BG contrast is relatively low with a complex background. Though our adopted pretreatment, steel bars can be segmented well and there still be a little noise that cannot be removed. It means that pretreatment we used may not work very well in some low FG and BG contrast environments but this hardly influenced subsequent processing. In general, the pretreatment we adopted can get good results under different conditions.

### 3.2. Steel Bar Boundary Localization Method

In general, the diameter is the closest inner distance of two thinned edges, and the distance is the closest outer edges distance of adjoining steel bars. However, it is easy incur in an error owing to the lack of precision on account of the discretized image, influence of noise, and jagged edges.

**Figure 3:**
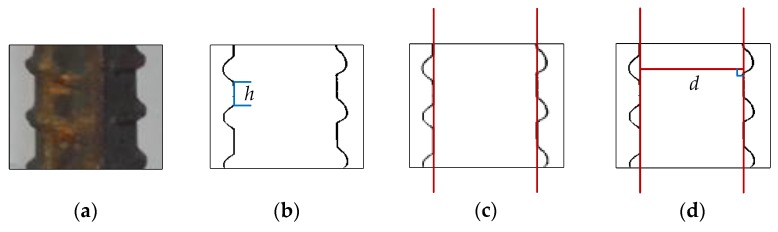

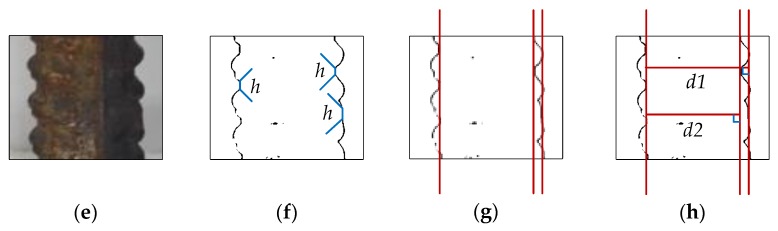
Two commonly edge detection state: (**a**) ideal state of steel bar; (**b**) apropos Hough transformation threshold; (**c**) right inner position acquired; (**d**) correct diameter obtained; (**e**) common state of steel bar; (**f**) another Hough threshold; (**g**) wrong inner position acquired; (**h**) erroneous result of diameter we may obtain.

Many common methods are used to detect lines in images [15,16,17,18,19], The Hough transform is one of those methods. When facing the problem of detecting steel bar lines, its results may not be rational in many conditions, an even erroneous. For instance, it’s the ideal situation we encounter from the processed images that steel bars are critically vertical (Figure 3a) and the threshold of Hough transform we set is apropos (Figure 3b), which gets the right inner position of the steel bars (red lines in Figure 3c), and we can get the right diameter, denoted by *d*, as shown in Figure 3d. However, the most common state we encounter is shown as Figure 3e. If we adjust the threshold (Figure 3f), we may obtain the outer position, nevertheless we cannot get each outer position, which is depicted as red lines in Figure 3g, and we will get wrong diameter *d*2 as the right diameter is denoted as *d*1 (Figure 3h). Hence, under the condition of a fixed threshold, it seems that this is an impossible task for the Hough transform algorithm. Similarly, other line detection algorithms are also confronted with such a dilemma.

In view of this, this paper proposes a sub-pixel boundary location method (SPBLM) based on the image projection. This method uses the directional projection of the acquired image. Then the image can be easily processed and we can obtain the information we require.

**Figure 4:**
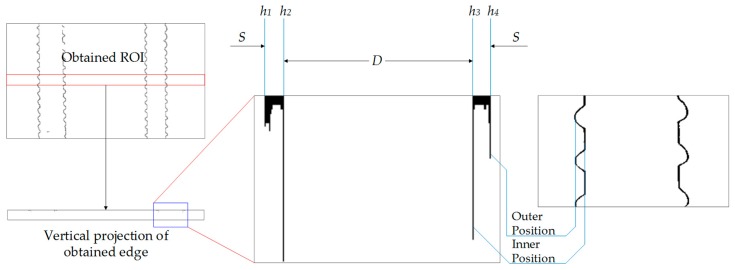
Schematic diagram of sub-pixel boundary positioning.

Figure 4 is a schematic diagram of sub-pixel boundary positioning by finding the projection edges. We give the pixel positions of four peaks of one steel bar as *h*_1_, *h*_2_, *h*_3_ and *h*_4_. Moreover, the diameter is denoted as *D* and spacing is given as *S*. The left two figures show the ROI (red rectangle), for decreasing calculation amount, and vertical projection of the edge-detected image processed by Canny operator, and the middle figure shows a partial enlargement of the vertical projection (blue rectangle). As can be seen from the right figure, each refinement boundary has two peaks (the position is indicated by the blue line), which can be seen as the outer position and inner position of saw tooth edge, compared with the right figure, a crop of the segmentation image.

**Figure 5:**
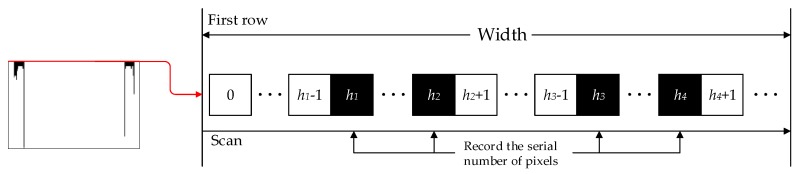
Recording the pixel numbers of edges by scanning the first row of pixels of a projection.

To fix the value of *h*_1_, *h*_2_, *h*_3_ and *h*_4_, we do a scan for the first row of pixels, as shown in Figure 5. Obviously, owing to the structural characteristics of steel bars, when the value of the pixel changes from 0 to 255 or in the opposite direction, the peak appears and we record the serial number of pixels as values of *h*_1_, *h*_2_, *h*_3_ and *h*_4_. Consequently, values of *h*_1_ and *h*_4_ are the outer position while values *h*_2_ and *h*_3_ are the inner position of each steel bar.

### 3.3. Steel Bar Dimension Calculation Method 

In this paper, the dimension calculation method is to transform the pixel difference to an actual size. After obtaining the value of *h*_1_ to *h*_4_, we can simply calculate the inner-inner position pixel difference $\delta_{in}$ and outer-outer position pixel difference $\delta_{out}$. Subsequently, it is necessary to multiply a converse parameter to get the real dimension. The equations can be defined as follows:

(1) $$\left\{ \begin{array}{l} D=\alpha \times \delta_{in}=\alpha \times \left|h_{3}-h_{2}\right|\\ S=\alpha \times \delta_{out}=\alpha \times \left|h_{1}-h_{4}\right| \end{array}\right.\;$$

where *h*_1_ and *h*_4_ should be obtained from two adjacent steel bars and *h*_1_ should be obtained from the right one while *h*_4_ should be obtained from the left one because of the scanning direction. α is the conversion parameter, transforming the pixel difference value to an actual size value. The diameter is given as *D* and the spacing is given as *S*.

Several methods have been proposed for the determination of α values. Due to the fact different phones have various lens focal lengths, and the presence of electronic image stabilization during recording which trims the picture, we do a test whose object is get the accurate value of α for almost situation when using a smartphone. We put the phone at a fixed height to shoot a scale in the middle of the frame, and it was set on the video mode with electronic image stabilization mode on. Then we intercept a frame from the video, and the number of pixels corresponding to different lengths at this height is obtained through processing. Subsequently, do same test under different fixed heights. Partial experimental data are shown in Table 3, including fixed height, scale length, number of pixels, and transformation parameter.

**Figure 6:**
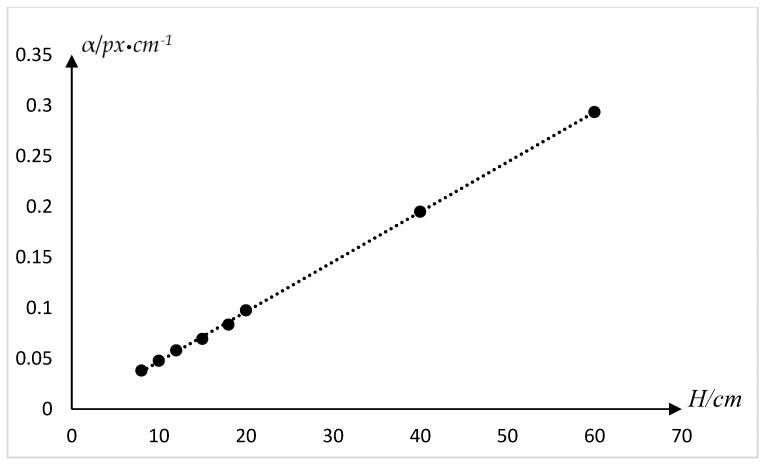
Pixel value/actual size transformation.

For the sake of showing the relationship between *H* and α more intuitively, we have drawn the following chart (Figure 6). A linear relationship has been found between *H* and α from the chart, then we make fitting calculation to obtain the experimental formula as follows:

(2) α = 0.0049H−0.0032 

In order to minimize the error caused by the viewing angle in video detection, detection and calculation are always performed on ROI when in between every two steel bars it moves to the center of the picture, namely calculating the average of the closest inner positions of two adjacent bars and it is approximately equal to the width of ROI, the result of that frame is the output.

### 3.4. Steel Bar Counting Method 

Since the processing of video will eventually be converted into image processing, it is necessary to use image stitching technology [20,21,22]. Image splicing is the technique of combining images with overlapping parts into a large, seamless and high-resolution image. There are many methods of image splicing, whose different algorithm steps may vary, but the general process is the same. We propose a fast stitch method (FSM) to record the last column pixels of each frame of the video and the last frame of the entire video by a matrix, and then the matrix is converted into a spliced image successfully. Suppose that the video input has a total of *n* frames, and each frame has i×j pixels, then the concrete steps are as follows:

Step 1: Record the pixel value of the last column of the first frame image as $\phi_{1i}$.
Step 2: Record the pixel value of the last column of the second frame image as $\phi_{2i}$, and obtain $\phi_{1i}+\phi_{2i}$.
Step 3: Record the pixel value of the last column of the (*n −* 1)-th frame image as $\phi_{(n-1)i}$, and obtain $\sum_{1}^{n-1}\phi_{i}$.
Step 4: Since only the last column of all the frames cannot contain all the bars, the entire image of the last frame is saved and spliced into the preceding sum, which is to obtain $\sum_{1}^{n-1}\phi_{i}+\phi_{n}$.

**Figure 7:**
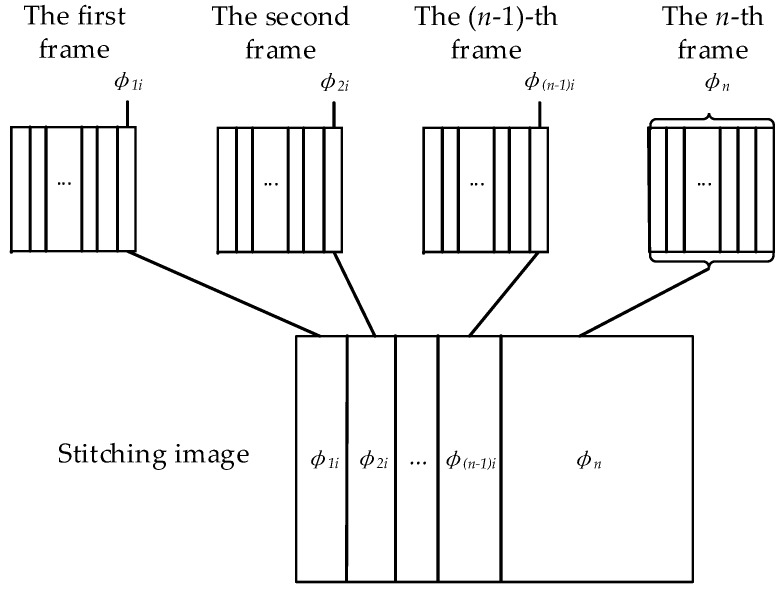
Image stitching treatment.

Among them, $\phi_{i}$ is the last column of pixel values for each frame of the image, then $\phi_{ki}$ is the pixel value of the last column of the *k*-th frame, k=1,2,…,n, $\phi_{n}$ is all pixel values of the last frame. Combined with Figure 7, the image stitching process can be more easily understood. Since only the last column of each frame will be processed during stitching, a correct and able-to-use image can be spliced as long as the shooting speed is not too fast.

**Figure 8:**
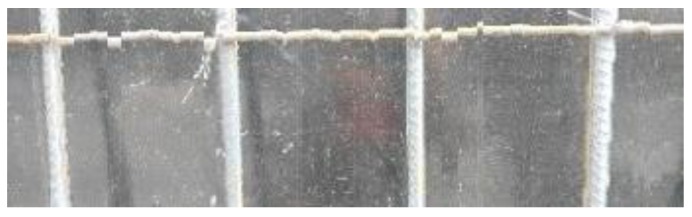
Part of one stitching image.

After obtaining the whole stitching image, we do the same treatment we proposed in Section 3.1 and Section 3.2, recording numbers of edges—four edges of every steel bar—denoted as *i*. Obviously, the number of steel bars is *i*/4. We get many stitching images at the construction site, and a part of one is as shown in Figure 8.

## 4. Components of the Steel Bars Position Quality Inspection System

### 4.1. Hardware

**Figure 9:**
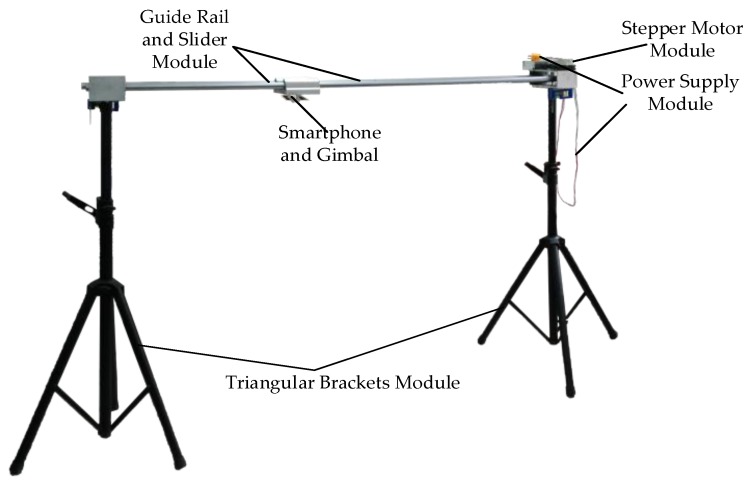
Overall structure diagram of the acquisition device.

As shown in Figure 9, the data acquisition device proposed in this paper mainly includes the following parts: two triangular brackets, a guide rail, a stepper motor with controller and driver, a DC power supply, a slider, a gimbal, and a smartphone. The acquisition device comprises a guide rail which is supported by a triangular bracket connected with both ends of the guide rail. And a slider is linked to the stepping motor which connected with its controller via its driver through the guide rail. The driver and controller are connected to power supply module. A smartphone for acquiring the image or video of the construction site is arranged on the slider through a gimbal.

#### 4.1.1. Main Hardware Components

**Figure 10:**
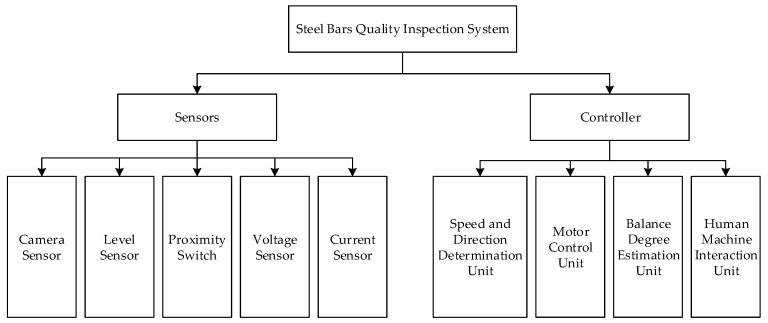
Main hardware components of the steel bars quality inspection system.

The main hardware components of acquisition device are presented in Figure 10. It can be seen that the steel bars quality inspection device in the construction site is primarily comprised of four types of sensors and a controller. These sensors include CMOS, level sensor, proximity switch, voltage sensor, and current sensor. The controller is the core for calculation and control of this inspection system, which mainly contains a speed and direction determination unit, motor control unit, balance degree estimation unit, and human machine interaction unit.

#### 4.1.2. Sensors

Sensors are employed to automatically and continuously detect the working status of the device on the construction site, thus appropriate ones with certain mechanical and electrical properties should be chosen to satisfy the actual measurement needs. Table 4 gives some key specifications of five types of sensors involved in this inspection system. To be specific, CMOS is used to capture images and videos. The level sensor is for its role in estimating the degree of balance of the whole device. The proximity switch is applied to make the slide stop automatically when it is close to both ends of the rail. The voltage sensor and current sensor are used to ensure a stable power supply for the whole system and steady the output of the stepper motor.

It is known that noise usually has negative impact on follow-up processing and analysis of signals, especially for weak analog signals. In this study, a porous metal shell has been used to shield noises during the analog signal measurement process.

#### 4.1.3. Controller

**Figure 11:**
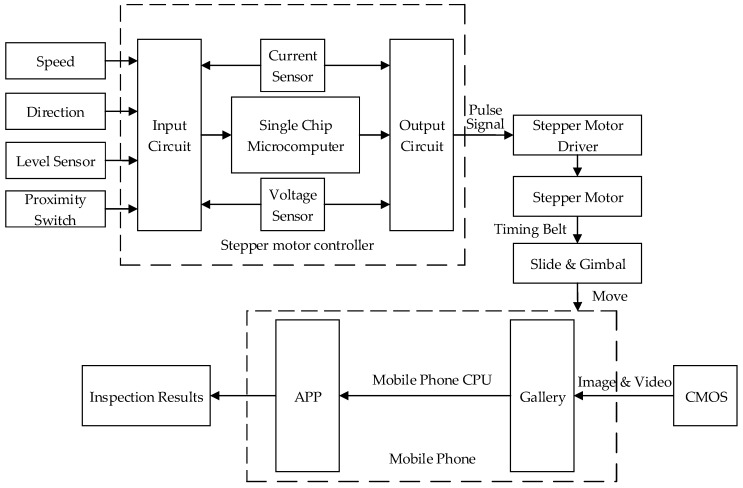
Functional block diagram of the controller.

**Figure 12:**
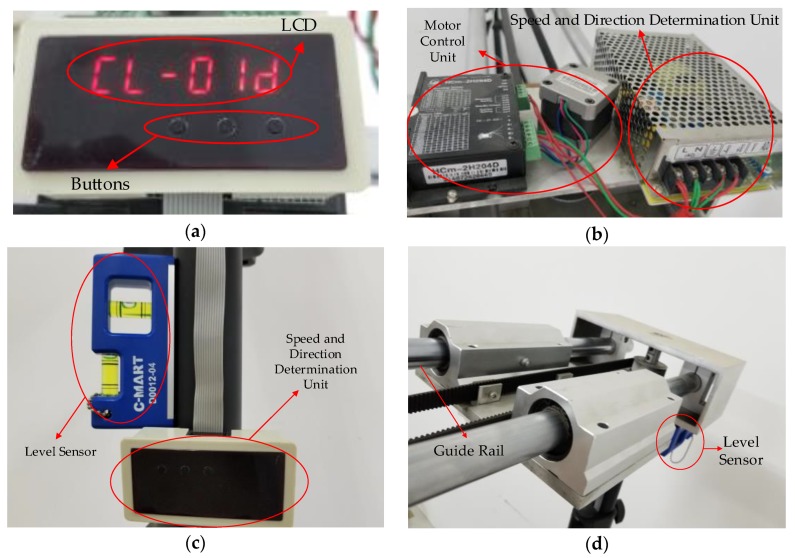
Physical appearance of sensors and controllers: (**a**) Panel of the controller; (**b**) fixed parts of the controller; (**c**) Panel of the controller and level sensor; (**d**) Guide rail and level sensor.

The controller in the construction site is the core hardware component of the inspection system, which can fulfill the tasks of speed adjustment and direction determination, balance degree estimation, and human machine interaction. Its functional block diagram and physical appearance can be found in Figure 11 and Figure 12, respectively. The following are the main functions and features of this controller:

- Speed adjustment and direction determination. This unit has been dedicated to set velocity and direction by inputting the voltage.
- Balance degree estimation and motor control. The balance degree estimation unit is devoted to ensure the shooting height is fixed. The motor control unit is used to adjust the motor speed flexibly by means of input value to make sure that gimbal, with the smartphone, is running at the optimal speed and decelerating when slider is close to both ends. In the controller, a single chip microcomputer has been designed to implement the tasks of speed and direction control, and proximity limit will give the signal to the microcomputer to determine when to decelerate.
- Human machine interaction. A LCD, a keyboard with three buttons on the panel, and the smartphone are used for the interaction between human and machine. A LCD is installed for displaying the current speed and direction of stepper motor. Buttons are mainly responsible for parameter (velocity & direction) setting and devoted to manual operation in an emergency, such as emergency stop, system reset, and power switch. The stepper motor will drive the timing belt and finally make the smartphone move to capture videos. After acquisition finishes, we uninstall the smartphone from the device and open the APP to process and get the inspection results.

### 4.2. Software

#### 4.2.1. Development Environment

**Figure 13:**
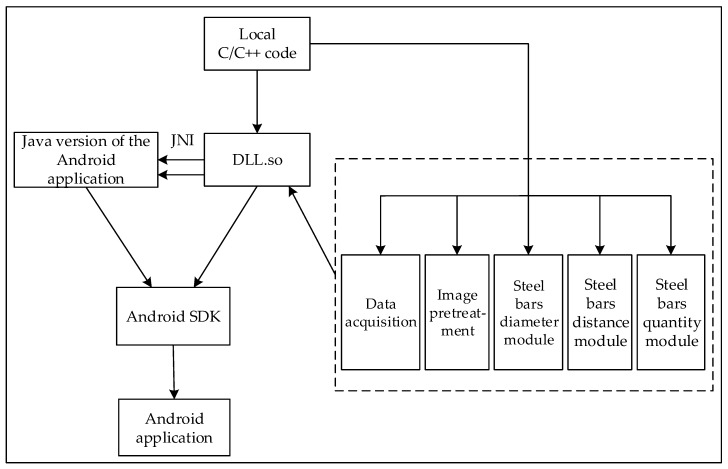
Overall system framework design block diagram.

The development process of this article mainly includes the algorithm design and simulation on Matlab, then debug in C code. Its functions include data acquisition, image pretreatment, modules of diameter and spacing measuring and number counting. Then it is ported to the Android Development Environment, while developing an Android host human-computer interaction interface. From the overall framework design of the system, it is divided into development of the local C/C++ code and the upper interface of Android human-computer interaction (developed by Java). These codes are first debugged on Qt platform and then packaged to satisfy JNI interface. NDK tool is used to compile and generate dynamic link libraries. Android application Java terminal includes a main window, a parameter setting module, and a detection module. The overall system framework design block diagram is shown in Figure 13.

#### 4.2.2. Development Platform

**Figure 14:**
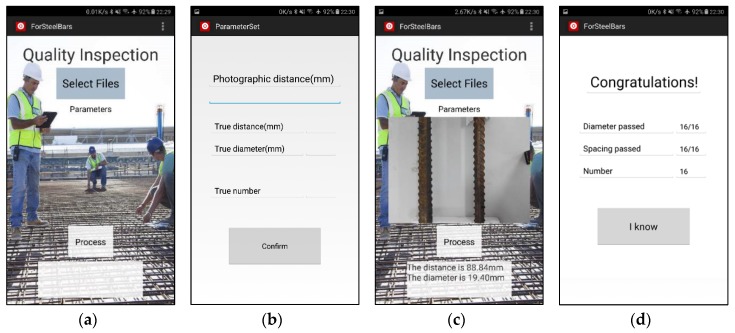
Android application interface: (**a**) Main interface; (**b**) Parameter setting interface; (**c**) Current processing results showing on main interface; (**d**) Final results interface.

Nowadays, Android smartphones occupy a large market share globally. The proportion of domestic Android system smartphones reaches up to 85%. When compared with PCs, smartphones are more portable. Moreover, with the rapid development of mobile phone performance, especially the growing performance of CPU/GPUs, smartphones carry more processing power than ever. As a result, more people start to enjoy dealing with their affairs on their phones, and Android smartphones are being used as testing platforms. Our Android application interface is shown in Figure 14.

When we open the APP, we can see several modules in the main interface (Figure 14a), including “Select Files” button, “Parameters” button, “Process” button, an image display area, and a result display area. We click “Select Files” button first to select an image or a video, and then we click “Parameters” button to turn to parameter setting interface to set photographic height (Figure 14b). The true diameter, spacing and number is not necessary to input if you just want to measure steel bars. After setting the photographic distance, we can do image process by clicking “Process” button (Figure 14c). The selected image or video has been shown in image display area and results have been shown in another area. In result display area, we give current diameter of the left one of two middle steel bars and spacing of two middle steel bars showed in image display area. At the end of process, we can see final results in another interface when we touch the result display area in main interface. In final results interface, for example, in Figure 14d, we can see that we process a video of 16 steel bars. The counting number is 16, and qualified numbers of steel bar (diameter and spacing) are all 16. The conclusion of inspection is “Congratulations!”

## 5. Results and Discussion

### 5.1. Statistical Results of Steel Bars Dimension Inspection

Using the inspection system developed in this paper, we will focus on static image and video processing results, mainly discuss detection results and their errors.

In the laboratory, we set the fixed height as 20 cm, and results are as shown in Table 5. Groups 1–3 are inspection results of static images and Groups 4, 5 are video results. In the right four columns, where the first and second value “number-D” represents the diameter of the left and right steel bar, and the third value “number-S” represents the spacing of two steel bars. We can see that the measured spacing value has a very low error compared to the true value in most instances, especially in static images and the result is a little bit higher in video detection. To be specific, in the former three groups, we placed two steel bars of the same or different diameter (20 mm/15 mm) at different spacings (105 mm and 120 mm). We do a static image process and get the result that the lowest error is 0.04 mm (absolute error) and 0.002% (relative error) in measuring diameter while the highest error is no more than 2.8 mm (absolute error) and 3.00% (relative error) in measuring spacing. This has reached a fairly low level, namely our device and method have a rather high accuracy in processing static images. In Groups 4 and 5, we take videos and test our method. Because video cannot be as clear as picture in the same state, results show that error is as high as 1.18 mm (absolute error) and 7.13% (relative error). However, it is still lower than some existing image processing method introduced in Section 1. More importantly, our method can do treatment and display the result in real time, which is not available in other methods.

At a construction site, we set the fixed height as 50 cm, and results are as shown in Table 6. All groups are video inspection results. As the results show, we can see that the absolute error and relative error are not high (1.66 mm/5.93%), which is as same as the result in laboratory. It can be sure that our device and method have a practical application to measure diameter and spacing of steel bars.

### 5.2. Statistical Results of Steel Bars Counting Number

Using the inspection system, we will only focus on video processing counting results. We set the fixed height as 100 cm (Groups 1, 3 and 4) and 80 cm (Groups 2 and 5) and collect videos at the construction site. Results are as shown in the Table 7. It is obvious that results of all groups are correct. For instance, the true number of steel bars in Group 1 is seven and the number counted through our method is the same. The count numbers are all correct for the other groups, too. This is a powerful support to say that our device and method can be used in actual environment to count numbers of steel bars.

### 5.3. Statistical Results of Processing Time

In this inspection system, we use a Samsung GALAXY S7 smartphone. It has an Exynos 8890 SoC (Octa-core@2.7 GHz) and a 4 GB LPDDR4 RAM (1866 MHz). For each photo (4083 × 3063, 12 MP) and videos (1080p@30 fps), the relevant processing time is as shown in the Table 8. We record the processing time of each photo and it is between 0.054 s and 0.067 s. Similar processing has been done with videos and we obtain an experimental formula that relates video time (VT) and processing time (PT):

(3) PT=0.5717×VT+1.929 (s) 

The formula means that it needs at least 1.929 s to stitch an image, and per additional second video needs extra 0.5717 s time cost. It can be seen from these two sets of data that proposed method can do real-time processing.

## 6. Conclusions

The presented quality inspection system for steel bars adopts advanced machine vision technology. It solves the real-time inspection problem of steel bars including diameter and spacing measuring and number counting. The research shows that machine vision can substitute most manual work in steel bars quality inspection, and furthermore, improve the production efficiency.

Compared with the existing devices, the proposed data acquisition device can be used to capture images and videos of banding steel mesh at construction sites, preparing them for later quality inspection. Each component is assembled, so it is convenient to disassemble and transport. The device can realize automatic collection of image and video of steel bars, and promotes the intelligent development of the modern construction industry, and has great application prospects.

Compared with the existing methods, the proposed method has a very low related error in static image processing when calculating diameter and spacing (as low as 0.04 mm (absolute error) and 0.002% (relative error)) and zero error in counting the number of steel bars.

The overall operation is simple and convenient, and the acquired image is clear. The device and method have a high adjustment precision, can realize real-time detection of steel construction quality which is unavailable in other existing methods. Moreover, it should be broadly applicable at various construction sites.

## Figures and Tables

**Table 1:** Articles about quality inspection of steel bar.

References	Measuring Parameters	Sensors	Inspection Method	Absolute Error	Relative Error
Defects detection methods of bar surface size under complex illumination [4]	Diameter	CCD	Machine vision	More than 0.072 mm	More than 0.18%
Application of ground penetrating radar in the survey of the rebar in concrete slab [5]	Diameter and spacing	Ground penetrating radar	Radar image processing	More than 2 mm	More than 10%
Application of wavelet transform in GPR to detect reinforcing bar [6]	Diameter and spacing	Ground penetrating radar	Wavelet transform	More than 0.4 mm	More than 1.6%
GPR measurement of the diameter of steel bars in concrete specimens based on the stationary wavelet transform [7]	Diameter and spacing	Ground penetrating radar	Radar image processing	More than 2 mm	More than 9.1%
Extracting dimensional information from steel reinforcing bars in concrete using neural networks trained on data from an inductive sensor [8]	Diameter and spacing	Inductive sensor	Neural networks	More than 0.12 mm	More than 1.1%
Method for detecting the diameter of steel bar in wall by terahertz wave or millimeter wave [9]	Diameter	Terahertz wave or millimeter wave	Measuring the power of reflected signal	-	-
Precise Diameter Measurement of Reinforcing Bar and Steel Pipe based on Bi-static Model using Microwave Radar [10]	Diameter	Microwave radar	Microwave propagation time	More than 5.6 mm	More than 20%

**Table 2:** Several results of pretreatment.

Group	Original Image	Image after Median Filter	Segmentation by Otsu Method	Edges Detected by Canny
1	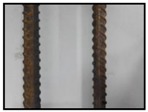	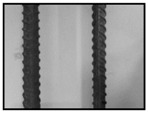	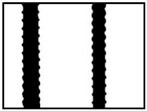	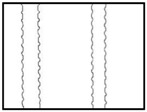
2	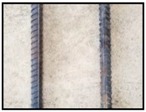	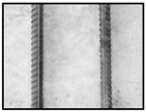	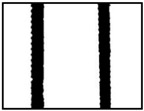	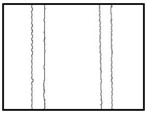
3	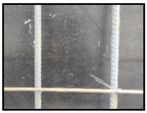	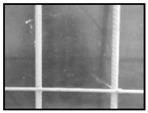	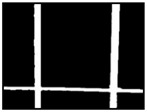	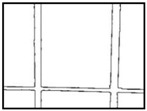
4	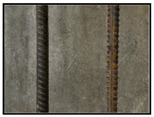	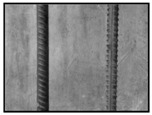	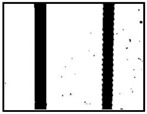	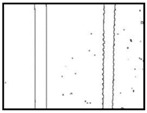

**Table 3:** Pixel value/actual size transformation.

Fixed Height *H* (cm)	Scale Length *D* (mm)	Number of Pixels (px)	Transformation Parameter α $(\mathrm{cm}\times {\mathrm{px}}^{-1})$
8	60	1575	0.038095238
10	80	1675	0.047761194
12	90	1550	0.058064516
15	100	1440	0.069444444
18	120	1438	0.083449235
20	140	1434	0.097629010
40	150	769	0.195058518
60	150	511	0.293542074

*H* is regarded as a fixed height, and *D* is the scale length. Parameter α is the quotient of scale length divided by the number of pixels.

**Table 4:** Specifications of the five types of sensors.

Parameter		Sensor			
	CMOS	Level Sensor	Proximity Switch	Voltage Sensor	Current Sensor
Measurement range	16 MP	0~90°	0~2.0 cm	0~40 V	0~2.21 A
Degree of precision	1 px	0.05°	0.1 cm	1.0%	1.0%
Temperature range	−20~50 °C	−5~70 °C	0~50 °C	−40~70 °C	−40~70 °C

**Table 5:** Results of true and measured values of diameter (-D) and spacing (-S) of steel bars in the laboratory.

Group	Collected Images/Video Screenshot	True Value (mm)	Measured Value (mm)	Absolute Error (mm)	Relative Error
1	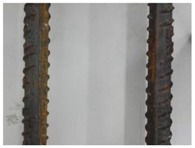	20.00-D 20.00-D 105.00-S	19.96-D 20.29-D 107.80-S	0.04-D 0.29-D 2.80-S	0.002%-D 1.45%-D 2.67%-S
2	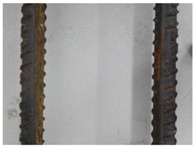	20.00-D 20.00-D 120.00-S	20.18-D 20.07-D 122.26-S	0.18-D 0.07-D 2.26-S	0.90%-D 0.35%-D 1.88%-S
3	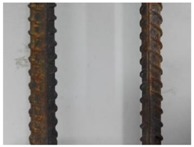	20.00-D 15.00-D 90.00-S	19.40-D 15.18-D 88.84-S	0.60-D 0.18-D 1.16-S	3.00%-D 1.20%-D 1.29%-S
4	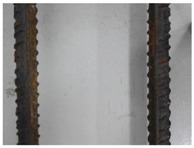	20.00-D 20.00-D 135.00-S	20.57-D 20.40-D 133.93-S	0.57-D 0.40-D 1.07-S	2.85%-D 2.00%-D 0.79%-S
5	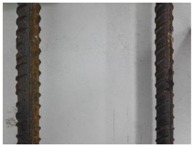	20.00-D 15.00-D 135.00-S	21.18-D 16.07-D 134.04-S	1.18-D 1.07-D 0.96-S	5.90%-D 7.13%-D 0.71%-S

**Table 6:** Results of true and measured values of diameter (-D) and spacing (-S) of steel bars at a construction site.

Group	Video Screenshot	True Value (mm)	Measured Value (mm)	Absolute Error (mm)	Relative Error
1	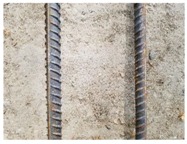	20.00-D 15.00-D 105.00-S	19.49-D 15.21-D 106.44-S	0.51-D 0.21-D 1.44-S	2.55%-D 1.40%-D 1.37%-S
2	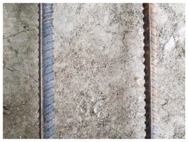	20.00-D 15.00-D 120.00-S	20.83-D 14.19-D 118.93-S	0.83-D 0.81-D 1.07-S	4.05%-D 5.40%-D 0.89%-S
3	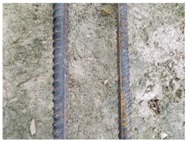	20.00-D 15.00-D 65.00-S	21.09-D 14.11-D 65.31-S	1.09-D 0.89-D 0.31-S	5.45%-D 5.93%-D 0.48%-S
4	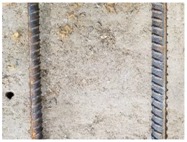	15.00-D 20.00-D 150.00-S	14.67-D 20.85-D 151.66-S	0.33-D 0.85-D 1.66-S	2.20%-D 4.25%-D 1.11%-S

**Table 7:** Comparison of True and Counting Number of Steel Bars.

Group	Frame of Video	True Number	Counting Number	Absolute Error	Relative Error
1	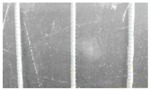	7	7	0	0
2	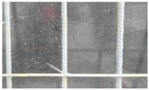	9	9	0	0
3	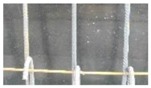	15	15	0	0
4	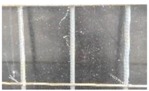	6	6	0	0
5	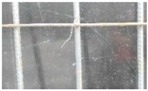	17	17	0	0

**Table 8:** Processing times of photos and videos.

Photo	Processing Time (s)	Video	Video Time (s)	Processing Time (s)
1	0.054	1	3.430	4.182
2	0.061	2	5.133	4.993
3	0.067	3	6.833	5.683
4	0.057	4	10.267	7.650
5	0.060	5	20.600	14.048

## Abbreviations

SPBLM	Sub-Pixel Boundary Location Method
FSM	Fast Stitch Method
